# Continuous adaptive radial sampling of k-space from real-time physiologic feedback in MRI

**DOI:** 10.1186/1532-429X-17-S1-P37

**Published:** 2015-02-03

**Authors:** Francisco Contijoch, Yuchi Han, Michael Hansen, Peter Kellman, Eugene Gualtieri, Mark Elliott, Sebastian Berisha, James J Pilla, Robert C Gorman, Walter R Witschey

**Affiliations:** 1 University of Pennsylvania, Philadelphia, PA, USA; 2 NIH, Bethesda, MD, USA; 3 Drexel University, Philadelphia, PA, USA

## Background

Clinical cardiac CINE MRI takes advantage of the periodic nature of heart motion to combine data from multiple heartbeats to improve image quality. For a number of cardiac MRI applications, including stress testing, arrhythmias, and measurement of pressure-volume relationships, there can be significant aperiodicity, leading to motion artifacts or contrast variation due to inconsistent data between heartbeats. To overcome this hurdle, we propose a method, Adaptive Real-time K-space Sampling (ARKS), for continuously adapting the MRI acquisition in response to a real-time physiologic feedback signal.

## Methods

In ARKS, a physiologic signal, such as the ECG waveform, is measured, and the most recent signal is continuously compared to the signal history to determine the occurrence of similar periods in the past. The k-space location of data obtained from previous periods is used to determine how subsequent data is acquired. We hypothesized that this approach would have near-optimal sampling properties for all time periods and adapt well in response to arrhythmias. 3-lead chest ECG data was collected from 10 normal subjects to investigate the k-space locations chosen for sampling by the algorithm. 40 seconds of bSSFP sampling was simulated at ARKS-based radial k-space locations by encoding images obtained using golden-angle radial MR data with synchronous ECG logging on a 1.5T Siemens Avanto scanner in one normal human subject and two patients with arrhythmias.

## Results

**Figure 1:**
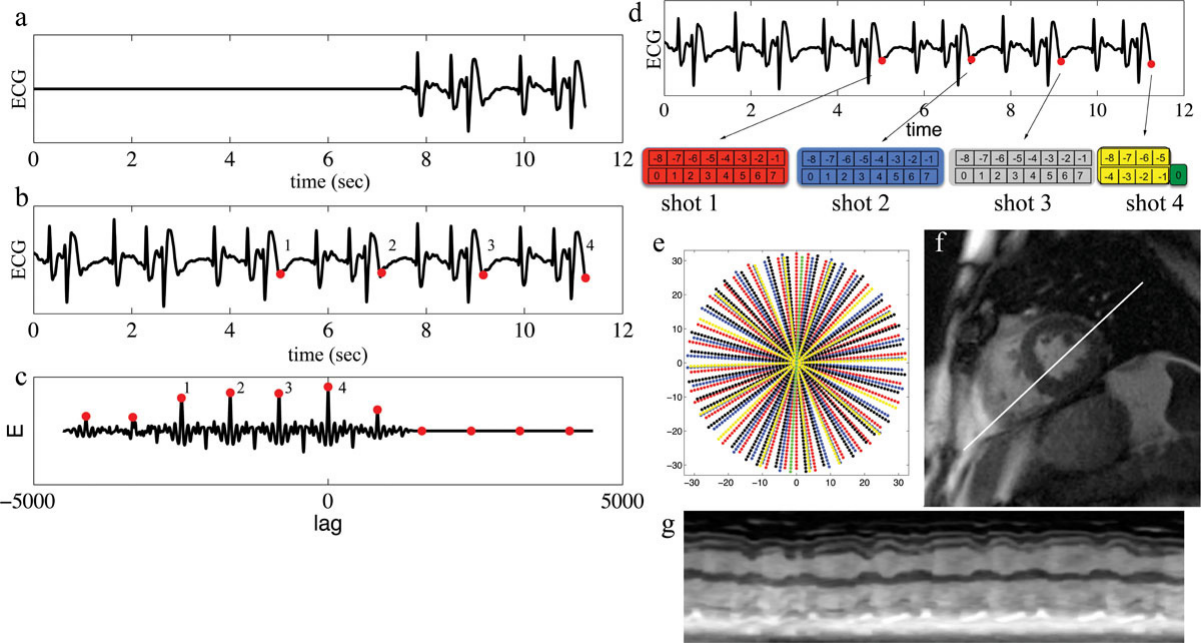
ARKS for a patient with a severe arrhythmia using a 4-16-57 scheme (shots-segments-projections). **A)** Signal buffer SS with the most recent physiologic data (1-2 heart beats). **B)** Large buffer SL storing history of the physiologic signal. 4 similar periods of the signal are labeled (red). **C)** Cross-correlation between SS and SL. Local maxima are labeled (red). Lags for the first 4 local maxima are used to label the ECG in **B**. **D)** For the first 3 shots, 16 radial projections are acquired and labeled -8 to 7, with the 0 projection corresponding to the lag index in **C**. 8 projections are acquired in the last shot. These 56 projections determine the angle of the 57th projection. **E)** 57 k-space radial projections. Projection color corresponds to the shot index in **D**. **F)**Simulated image from radial projections prescribed in **E**. **G)** Time-varying ventricular volume from the line in **F**.

Across several combinations of shots (number of previous periods) and segments (radial projects per period), ARKS resulted in higher sampling uniformity than random radial sampling as well as golden-angle sampling. An example of ARKS sampling for a subject with a regular arrhythmia is shown in Fig 1. The rhythm in this subject featured a normal beat, a second beat interrupted by a pre-ventricular contraction, followed by a third long beat with a longer RR-interval. Each of the three beats were correctly differentiated and segmented for ARKS, despite distortion of the ECG waveform by the electrohemodynamic effect of the MRI scanner. Simulated ARKS images correctly depicted the tri-beat arrhythmia morphology in real-time (Fig. 1F).

## Conclusions

Adaptive real-time k-space sampling is a near-optimal sampling scheme that shares the benefits of real-time display, improves the signal-to-noise and artifact levels of a multi-shot radial k-space trajectory and, by adapting to a physiologic signal, mitigates deleterious motion artifacts. We anticipate that this approach to k-space sampling would have benefits for clinical examinations of the heart.

## Funding

The authors would like to thank the National Institutes of Health National Heart Lung and Blood Institute for support through grants R00-HL108157, R01-HL063954 and HL-073021.

